# DNA minicircles capable of forming a variety of non-canonical structural motifs

**DOI:** 10.3389/fchem.2024.1384201

**Published:** 2024-03-26

**Authors:** Lukáš Trizna, Jakub Olajoš, Viktor Víglaský

**Affiliations:** Department of Biochemistry, Institute of Chemistry, Faculty of Sciences, P. J. Šafárik University, Košice, Slovakia

**Keywords:** non-canonical motifs, G-quadruplex, i-motif, circular dichroism, TGGE, DNA minicircle

## Abstract

Although more than 10% of the human genome has the potential to fold into non-B DNA, the formation of non-canonical structural motifs as part of long dsDNA chains are usually considered as unfavorable from a thermodynamic point of view. However, recent experiments have confirmed that non-canonical motifs do exist and are non-randomly distributed in genomic DNA. This distribution is highly dependent not only on the DNA sequence but also on various other factors such as environmental conditions, DNA topology and the expression of specific cellular factors in different cell types. In this study, we describe a new strategy used in the preparation of DNA minicircles containing different non-canonical motifs which arise as a result of imperfect base pairing between complementary strands. The approach exploits the fact that imperfections in the pairing of complementary strands thermodynamically weaken the dsDNA structure at the expense of enhancing the formation of non-canonical motifs. In this study, a completely different concept of stable integration of a non-canonical motif into dsDNA is presented. Our approach allows the integration of various types of non-canonical motifs into the dsDNA structure such as hairpin, cruciform, G-quadruplex and i-motif forms but also combinations of these forms. Small DNA minicircles have recently become the subject of considerable interest in both fundamental research and in terms of their potential therapeutic applications.

## Graphical Abstract

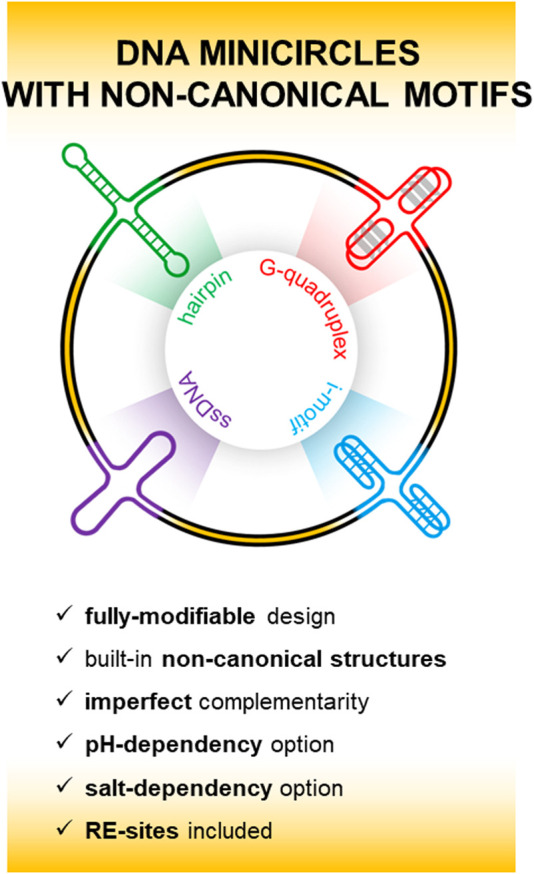

## 1 Introduction

Sequences that are capable of forming non-canonical structural motifs (NCMs) have attracted increasing scientific interest in recent years due to their importance in key cellular regulation processes. It is now generally accepted that non-canonical motifs play a significant role in controlling gene expression and are also involved in determining the overall fate of cells (Bansal et al., 2022; Makova and Weissensteiner, 2023). Recent studies have shown that NCMs, primarily G-quadruplexes, are ubiquitous in all spheres of life; they have been found in viruses, bacteria, unicellular eukaryotes, fungi, plants and animals (Tlučková et al., 2013; Métifiot et al., 2014; Marsico et al., 2019; Puig-Lombardi et al., 2019; Saranathan and Vivekanandan, 2019; Gazanion et al., 2020; Bohálová et al., 2021; Cantara et al., 2022), and it is also estimated that approximately 13% of the human genome has the capacity to fold into non-B DNA (Guiblet et al., 2018). The strong conservation of these secondary structures across evolution indicates that they fulfil important genome regulatory functions (Puget et al., 2019). The potential to adopt various NCMs is dependent on many factors, including nucleotide sequence, ionic strength, hydration and the presence of ligands. A- and B-DNA are the most widely studied forms of DNA, but other NCMs such as Z-DNA, hairpins/cruciforms, triplexes, G-quadruplexes and i-motifs have also been examined in detail (Pandya et al., 2021). NCMs can form spontaneously under certain conditions, but their occurrence as part of a dsDNA chain, such as a chromosome, is not so straightforward, and it is usually deemed more appropriate to use a simplified DNA model system for their subsequent analysis. In contrast to the double helix, the formation of NCMs in the dsDNA system is usually thermodynamically unfavorable, requiring the energy barrier to be overcome and somehow forcing dsDNA to form ssDNA, at least temporarily, in order to favor the formation of non-canonical motifs. The transient localized occurrence of single-stranded DNA in cells primarily appears during the processes of DNA replication, transcription and recombination (Puget et al., 2019); for example, the formation of cruciform and Z-DNA motifs requires a negative superhelicity value which contributes to their stabilization (Zheng et al., 1991). Similarly, G-quadruplexes are most likely to form when DNA is in the single-stranded state. In this study, however, we describe an alternative way of facilitating the embedding of NCMs in dsDNA.

DNA minicircles (MCs) are one of the simplest rigid objects at the nanometer scale. Small DNA circles were first prepared by designing two 21-mer DNA precursor sequences which, upon hybridization and ligation, resulted in a statistical distribution of DNA minicircles (Ulanovsky et al., 1986). Synthetic double-stranded MCs are attractive nano-objects that have received considerable attention due to their potential for use in therapeutic applications. DNA minicircles mimic the circular DNA found in various living organisms or in viruses. In the field of DNA nanotechnology, small MCs have been employed as building blocks to assemble nanoarchitectures after the incorporation of non-canonical motifs or chemically functionalized oligodeoxynucleotides, with this approach leading to the possibility of creating molecular devices with a broad range of functions (Mayer et al., 2008; Rasched et al., 2008; Gonçalves et al., 2010; Thibault et al., 2017).

In this study, we exploit the fact that imperfections in the pairing of complementary strands thermodynamically weaken the dsDNA structure at the expense of enhancing the formation of NCMs. The report describes an original versatile plasmid-free method for the preparation of DNA minicircles which can facilitate the formation of non-canonical motifs. We have designed a linear system consisting of a double-stranded oligonucleotide with introduced pairing imperfections; this basic building block features cohesive substrate ends and can efficiently generate minicircles in circularization reactions mediated by ligase. In addition, imperfections are the source of a certain degree of bending in the DNA strands which results in a limited number of closed circles in terms of the number of building blocks.

## 2 Materials and methods

All oligonucleotides were purchased from Metabion GmbH (Germany) as high-performance liquid chromatography (HPLC)-purified desalted samples and were used without further purification, scale was 0.02 µmol. The sequence of the central variable regions in the DNA oligonucleotides used in this study is shown in Table 1.

**TABLE 1:** Sequences and building blocks used in this study.

MCs	Building block of MC			Supported motif
	Abbrev	Sequence orientations: Up: 5`→ 3′ and down: 3`→ 5′	nts	
dsHTR (AT ends)	+HTR	AAA​AAA​AGA​TCT​GGG​TTA​GGG​TTA​GGG​TTA​GGG​CTG​CAG​AA	41	dsDNA
	-HTR	TTT​CTA​GAC​CCA​ATC​CCA​ATC​CCA​ATC​CCG​ACG​TCT​TTT​TT	41	
dsHTR-gc (GC ends)	+HTR^gc^	GCG​CGC​AGA​TCT​GGG​TTA​GGG​TTA​GGG​TTA​GGG​CTG​CAG​GC	41	
	-HTR-gc	CGT​CTA​GAC​CCA​ATC​CCA​ATC​CCA​ATC​CCG​ACG​TCC​GCG​CG	41	
ds-cMyc	+cMYC	AAA​AAA​AGA​TCT​TGG​GGA​GGG​TGG​GGA​GGG​TGG​GGC​TGC​AGA​A	43	
	-cMYC	TTT​CTA​GAA​CCC​CTC​CCA​CCC​CTC​CCA​CCC​CGA​CGT​CTT​TTT​T	43	
G_3_T/C_3_A	G3T	AAA​AAA​AGA​TCT​GGG​TGG​GTG​GGT​GGG​CTG​CAG​AA	35	
	C3A	TTT​CTA​GCC​CAC​CCA​CCC​ACC​CGA​CGT​CTT​TTT​T	35	
A/T	A_20_	AAA​AAA​AGA​TCT​AAA​AAA​AAA​AAA​AAA​AAA​AAC​TGC​AGA​A	40	
	T_20_	TTT​CTA​GAT​TTT​TTT​TTT​TTT​TTT​TTT​TGA​CGT​CTT​TTT​T	40	
AT/TA	(AT)_10_	AAA​AAA​AGA​TCT​ATA​TAT​ATA​TAT​ATA​TAT​ATC​TGC​AGA​A	40	
	(TA)_10_	TTT​CTA​GAT​ATA​TAT​ATA​TAT​ATA​TAT​AGA​CGT​CTT​TTT​T	40	
ATA/TAT	ATA	AAA​AAA​AGA​TCT​ATA​CTG​CAG​AA	23	
	TAT	TTT​CTA​GAT​ATG​ACG​TCT​TTT​TT	23	
GC/CG	(GC)_10_	AAA​AAA​AGA​TCT​GCG​CGC​GCG​CGC​GCG​CGC​GCC​TGC​AGA​A	40	
	(CG)_10_	TTT​CTA​GAC​GCG​CGC​GCG​CGC​GCG​CGC​GGA​CGT​CTT​TTT​T	40	
+/+ HTR	+HTR	AAA​AAA​AGA​TCT​GGG​TTA​GGG​TTA​GGG​TTA​GGG​CTG​CAG​AA	41	G4
	+HTR	TTT​CTA​GAG​GGA​TTG​GGA​TTG​GGA​TTG​GGG​ACG​TCT​TTT​TT	41	
+HTR/TAT	+HTR	AAA​AAA​AGA​TCT​GGG​TTA​GGG​TTA​GGG​TTA​GGG​CTG​CAG​AA	41	
	TAT	TTT​CTA​GA-------T--A--T-------GAC​GTC​TTT​TTT	23	
+HTR/T	+HTR	AAA​AAA​AGA​TCT​GGG​TTA​GGG​TTA​GGG​TTA​GGG​CTG​CAG​AA	41	
	T_20_	TTT​CTA​GAT​TTT​TTT​TTT-TTT​TTT​TTT​TGA​CGT​CTT​TTT​T	40	
+HTR-gc/T	+HTR^gc^	GCG​CGC​AGA​TCT​GGG​TTA​GGG​TTA​GGG​TTA​GGG​CTG​CAG​GC	41	
	a-T_20_	CGT​CTA​GAT​TTT​TTT​TTT-TTT​TTT​TTT​TGA​CGT​CCG​CGC​G	40	
+cMYC/T	+cMYC	AAA​AAA​AGA​TCT​TGG​GGA​GGG​TGG​GGA​GGG​TGG​GGC​TGC​AGA​A	43	
	T_20_	TTT​CTA​GA-TTT​TTT​TTT​T-TTT​TTT​TTT​T-GAC​GTC​TTT​TTT	40	
G_3_T/T	G3T	AAA​AAA​AGA​TCT-GGG​T-GGG​T-GGG​T-GGG-CTG​CAG​AA	35	
	T_20_	TTT​CTA​GAT​TTT​TTT​TTT​TTT​TTT​TTT​TGA​CGT​CTT​TTT​T	40	
−/− HTR	-sHTR	AAA​AAA​AGA​TCT​CCC​AAT​CCC​AAT​CCC​AAT​CCC​CTG​CAG​AA	41	iM
	-HTR	TTT​CTA​GAC​CCT​AAC​CCT​AAC​CCT​AAC​CCG​ACG​TCT​TTT​TT	41	
A/-HTR	A_20_	AAA​AAA​AGA​TCT​AAA​AAA​AAA​A-AAA​AAA​AAA​ACT​GCA​GAA	40	
	-HTR	TTT​CTA​GAC​CCT​AAC​CCT​AAC​CCT​AAC​CCG​ACG​TCT​TTT​TT	41	
A/-cMYC	A_20_	AAA​AAA​AGA​TCT-AAA​AAA​AAA​A-AAA​AAA​AAA​A-CTG​CAG​AA	40	
	-cMYC^iM^	TTT​CTA​GAA​CCC​CTC​CCA​CCC​CTC​CCA​CCC​CGA​CGT​CTT​TTT​T	43	
A/C_3_A	A_20_	AAA​AAA​AGA​TCT​AAA​AAA​AAA​AAA​AAA​AAA​AAC​TGC​AGA​A	40	
	C_3_A	TTT​CTA​G--CCC​ACC​CAC​CCA​CCC---GAC​GTC​TTT​TTT	35	
+/+ HP	HP1	GCG​CGC​AGA​TCT​AAA​AAA​CCC​TTT​TTT​CTG​CAG​GC	35	hairpin
	HP2	CGT​CTA​GAA​AAA​AAC​CCT​TTT​TTG​ACG​TCC​GCG​CG	35	

### 2.1 MC preparation and PAGE separation

**FIGURE 1:**
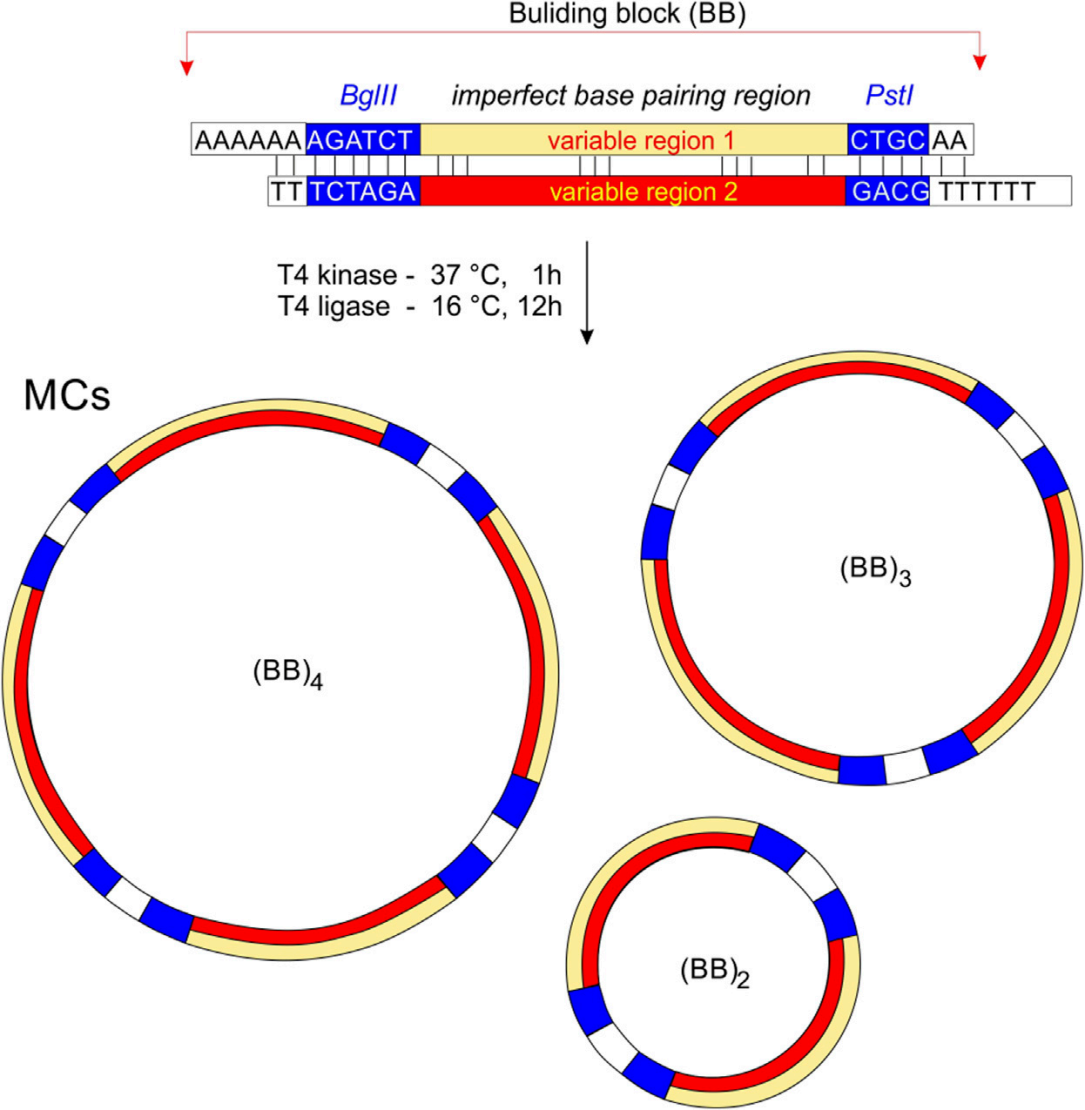
The principle of MCs preparation. Building bricks (BBs) are connected by cohesive ends to produce a series of MCs of different sizes. Non-perfectly complementary variable regions are highlighted in yellow and red. We assume that the minimum for the circularization is two building blocks, and the maximum depends on the sequence and condition of DNA ligation.

A schematic representation of the preparation of MCs is shown in Figure 1. Each oligonucleotide was phosphorylated at the 5′end prior to hybridization with T4 polynucleotide kinase (New England Biolabs) according to the manufacturer’s standard protocol. For the standard annealing process, the DNA strands were mixed in a hybridization buffer (70 mM Tris-HCl with 10 mM MgCl_2_, 5 mM DTT, pH 7.6) and heated at 95° for 10 min before being slowly cooled to room temperature. T4 DNA ligase and ligation buffers were purchased from New England Biolabs (Germany). DNA ligations were performed in 20 µL with 10 U T4 ligase for 10–20 h at 15°C. Heat inactivation of the ligase was then performed by heating the sample at 65° for 10 min and leaving it to cool to room temperature. The mixture was precipitated with two volumes of ethanol, dried and solubilized in sample buffer.

The ligation products were separated using nondenaturing PAGE in a temperature-controlled electrophoretic apparatus (Z375039-1 EA; Sigma-Aldrich, San Francisco, CA, USA) on 8% acrylamide (19:1 acrylamide/bisacrylamide) gels. The modified Britton–Robinson buffer (mBR) used TRIS instead of potassium/sodium hydroxide: 25 mM phosphoric acid, 25 mM boric acid and 25 mM acetic acid. 1 mM MgCl2 was also added in cases where 50 mM LiCl was present. The mRB was also used for spectral analyses and supplemented with either 50 mM potassium, sodium or lithium chloride; pH was adjusted with TRIS to a final value of 7.4, while acidic conditions (pH 4–6) were used for the proposed adoption of i-motif. Electrophoresis was run at 10°C for 3 h at 125 V (∼8 V⋅cm^-1^). A 20 bp ladder purchased from New England Biolabs (Germany) was used for all electrophoretic measurements. Each gel was stained with Stains-all (Sigma-Aldrich) and photographed with a Canon 3,000 digital camera. The DNA sequences used in this study are shown in Table 1.

### 2.2 TGGE

Temperature gradient gel electrophoresis (TGGE) equipment was used according to a method which has been described previously (Bauer et al., 2011). The gel concentration was 8%. Electrophoreses were run perpendicularly to the temperature-gradient (20°C–80 °C) for 4 h at 160 V (∼8 V·cm^−1^). Approximately 3–10 μg of DNA was loaded into the electrophoretic well. DNA oligomers were visualized with Stains-all after the electrophoresis.

### 2.3 Circular dichroism spectroscopy

CD and UV/Vis spectra were recorded on a Jasco J-810 spectropolarimeter equipped with a PTC-423L temperature controller using a quartz cell of 1 mm optical path length in a reaction volume of ∼150 μL; instrument scanning speed of 100 nm/min, 1 nm pitch and 1 nm bandwidth with a response time of 2 s over a wavelength range of 220–320 nm. All the CD spectra are baseline-corrected for signal contributions caused by the buffer. The CD melting analysis represents three averaged scans taken at temperatures ranging from 0°C to 100°C. All other parameters and conditions were the same as those which were described previously (Tóthová et al., 2014). Differential CD spectra were obtained by subtracting spectra under conditions that promote NCM formation from those under conditions that hinder NCM formation; for example, potassium or lithium ions were present for the G-quadruplexes, while acidic and neutral/basic pH values were used for the i-motif.

CD melting profiles were collected at ∼295 and ∼265 nm as a function of temperature using a procedure which has been published previously (Tóthová et al., 2014; Trizna et al., 2023).

### 2.4 TDS

Thermal Difference Spectra (TDS) were obtained by subtracting the UV absorbance spectra of the unfolded form at 95°C from the folded form at 20°C in given sample yield profiles that are characteristic of G4 (i.e., featuring a negative peak at 295 nm and a positive peak at 275 nm) (Mergny et al., 2005; Krafčíková et al., 2017).

### 2.5 Evidence of circularization by the terminal transferase

Extending the DNA chain by adding nucleotides to the free ends with DNA terminal transferase allows circular DNA to be distinguished from non-circular structures. If the DNA is not circularized, it displays free ends. These ends can be extended with a terminal transferase, but the circular DNA does not change in length when this enzyme is used. The presence of residual non-circular MCs after ligation may adversely affect the results. To prevent this artifact, we eluted the most intense electrophoretic bands (∼100 and ∼200 bp) from the gel and treated them with terminal transferase. The terminal transferase purchased from New England Biolabs (cat. no. M0315S) was used for this set of experiments according to the manufacturer’s standard protocol. The reaction was carried out at 37°C for 30 min at a volume of 20 µL.

## 3 Results

### 3.1 MCs capable of forming G-quadruplexes and i-motifs

**FIGURE 2:**
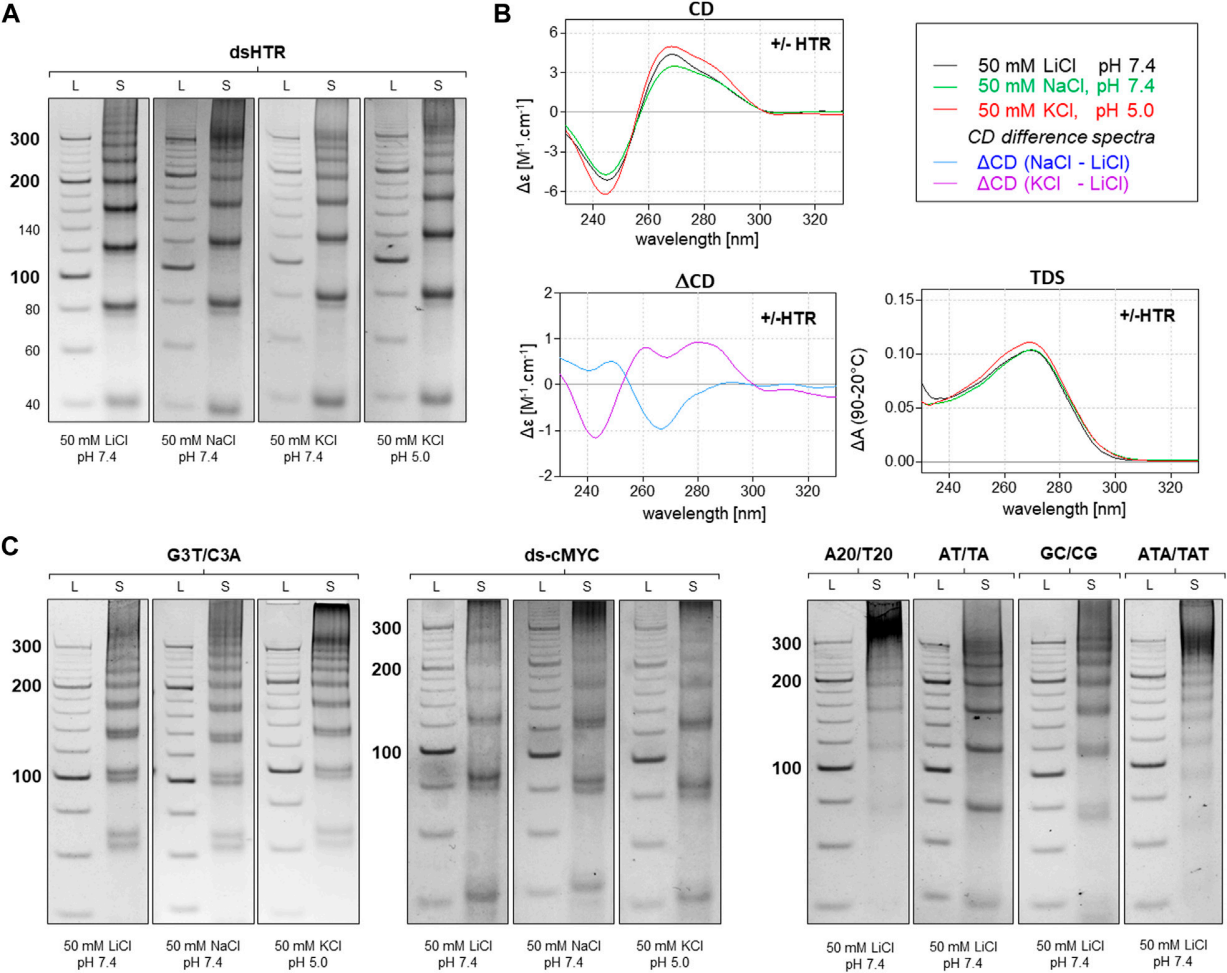
Analysis of MCs consisting of perfectly complementary building blocks. The electrophoretic separation of dsHTR MC under various ionic and pH conditions is presented in **(A)**. The symbol S and L represent the sample and 20 bp ladder, respectively. No significant difference in the mobility of MCs can be observed under any of the studied conditions. CD (20°C), ΔCD and TDS (95°C–20°C) under the same conditions are shown in **(B)**. All six additional samples showed the same effect as dsHTR **(C)**.

A variety of methods, including electrophoresis, CD, and UV-Vis absorption spectroscopy, were used to analyze the resulting DNA structures. The identified MCs consisting of DNA oligonucleotides which are separately capable of adopting non-canonical motifs, G-quadruplexes and i-motifs are shown in Figure 2. This set of MCs are assembled from building blocks that are perfectly complementary to each other, but even under conditions which are suitable for the adoption of G-quadruplexes and i-motifs, no difference in mobility was observed at pH five and seven in the presence of potassium ions. The same effect was observed with any perfectly complementary building blocks consisting of sequences either capable or incapable of adopting other structures than dsDNA, with no difference in mobility observed under various conditions. These results indicate that the formation of NCMs in MCs using complementary building blocks is thermodynamically very unfavorable.

**FIGURE 3:**
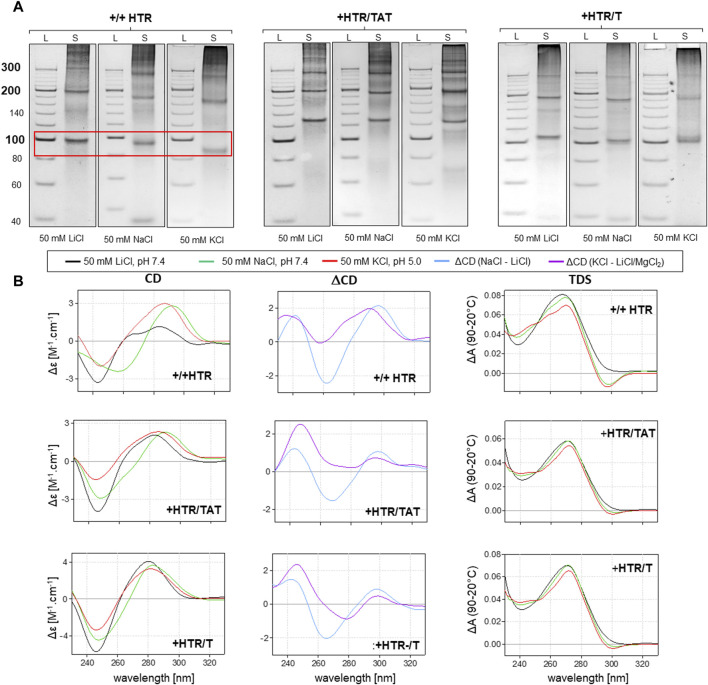
Analysis of MCs capable of adopting G-quadruplex forms. The electrophoretic separation of three different samples is presented in **(A)**. A clear difference in mobility can be observed in all the examples; the red rectangle is highlighted. The corresponding CD spectra, difference spectra and TDS under the same conditions are shown in panel **(B)**.

However, any imperfection in the complementarity of building blocks was found to contribute significantly to facilitating the adoption of NCMs (Figure 3). Anomalies in the MC mobility of +/+HTR, +HTR/TAT, +HTR/T, +HTR-gc/T, +cMyc/T and G3T/T suggest the adoption of G-quadruplex structures in the MCs. The electrophoretic mobilities of MCs were also found to differ from those of perfectly complementary MCs (Figure 3). Clear differences in mobility were also observed under conditions which are favorable for the formation of G-quadruplexes but also under those which are unfavorable. These findings are also supported by other measurements, such as CD and TDS, Figure 2B; Figure 3B. The results of the differential CD spectra suggest that the G-quadruplex in MC HTR adopts an antiparallel form in the presence of sodium and a hybrid form in the presence of potassium ions. The TDS spectra show clear negative peaks at 295 nm in the presence of potassium and sodium ions, a typical signature of the formation of G-quadruplexes.

**FIGURE 4:**
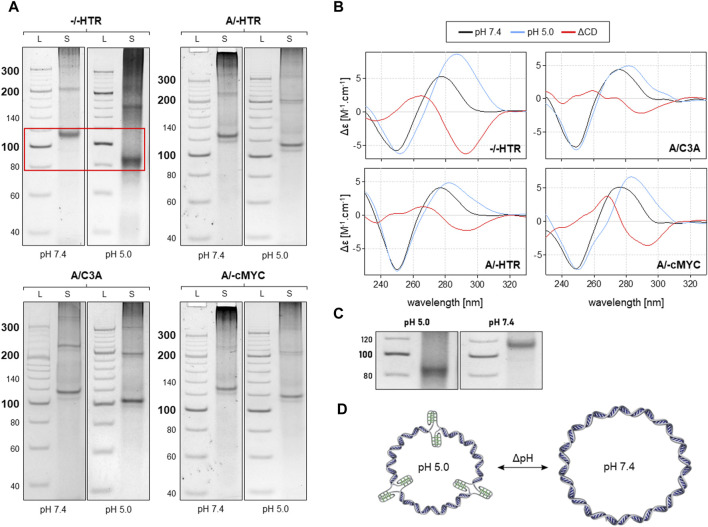
Analysis of MCs capable of adopting the i-motif. The electrophoretic separation of four different samples is presented in **(A)**. A clear difference in mobility can be observed in all the examples; the red rectangle is highlighted in the **(C)**. The corresponding CD spectra under the same conditions are shown in panel **(B)**. The proposed model of pH-dependent i-motif in MC for three building blocks is shown in **(D)**.

As can be seen in Figure 4, the MCs of −/−HTR, A/-HTR, A/-cMyc and A/C3A are capable of adopting i-motifs. The mobility of an individual band representing a specific MC is different at pH 7.4 and at pH 5.0. When an NCM is formed in the MC, the friction coefficient of the molecule in the electrophoretic gel is lower due to its more compact structure, thereby resulting in a slightly higher mobility. These results are also confirmed by CD measurements taken under the same conditions. Additionally, the results suggest that circularization is only preferred in the case of specific numbers of individual blocks, a feature which contrasts with that of non-NCM forming MCs where a regular increase in molecular weight was observed. This effect may be due to the deformation of the building blocks caused by imperfect base pairing; it is highly likely that some bending occurs under these conditions. This type of deformation would prevent the rings from closing by ligation with a specific number of building blocks.

While the linear ladder may not provide an accurate estimation of the size of MCs, combining it with a ladder composed of circular DNAs can improve accuracy. On the other hand, the electrophoreses shown in Figure 2 indicate that the ladder used may be accurate enough to estimate the size of MCs incapable of forming NCs.

### 3.2 Verification of MC circularization

**FIGURE 5:**
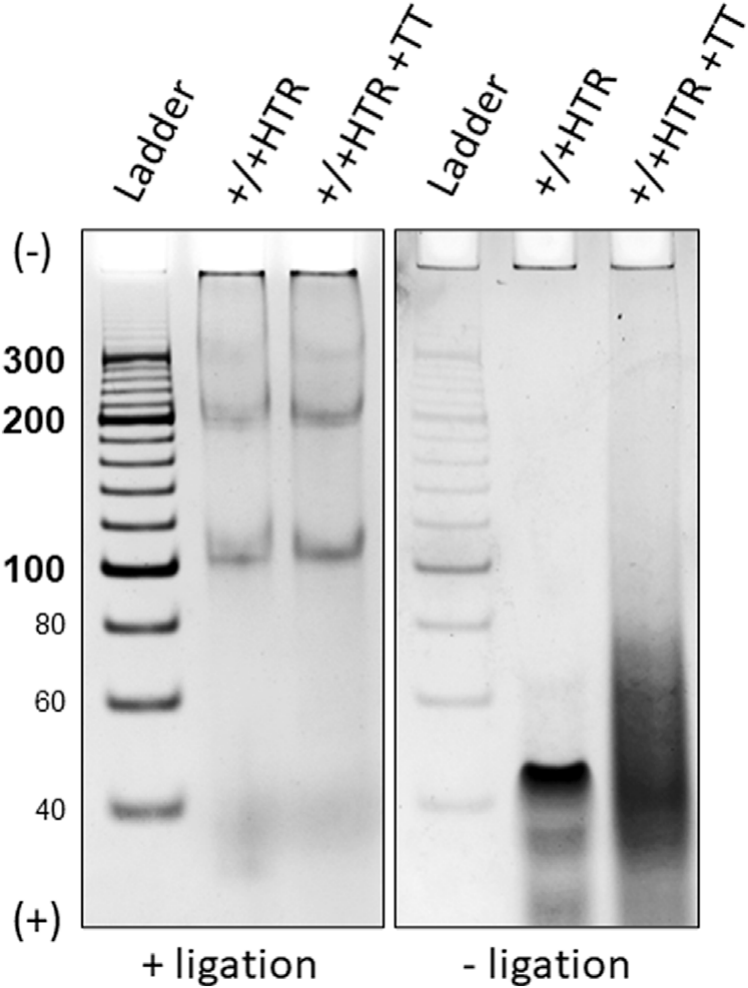
Electrophoretic separation of ligated and non-ligated building blocks treated with a DNA terminal transferase, left and right panels. Only two bands eluted from were used for this proof. The building blocks that did not terminate with ligase show a clear difference after treatment with terminal transferase (right), whereas no difference is observed for closed MCs (left).

A simple verification of the circularity of the MCs was performed using a technique which is described in the Methods section. Figure 5 shows the representative results of terminal transferase applied to the hybridized +/+ HTR building block and the MCs formed after ligation in the right and left panels, respectively.

Due to the absence of free ends in the MCs, no enzymatic elongation of the building blocks was observed after ligation, but the presence of a smeared band in the electrophoretic gel of non-ligated building blocks indicates its elongation. Identical results were obtained for each pair of oligonucleotides from which the MCs were constructed.

**FIGURE 6:**
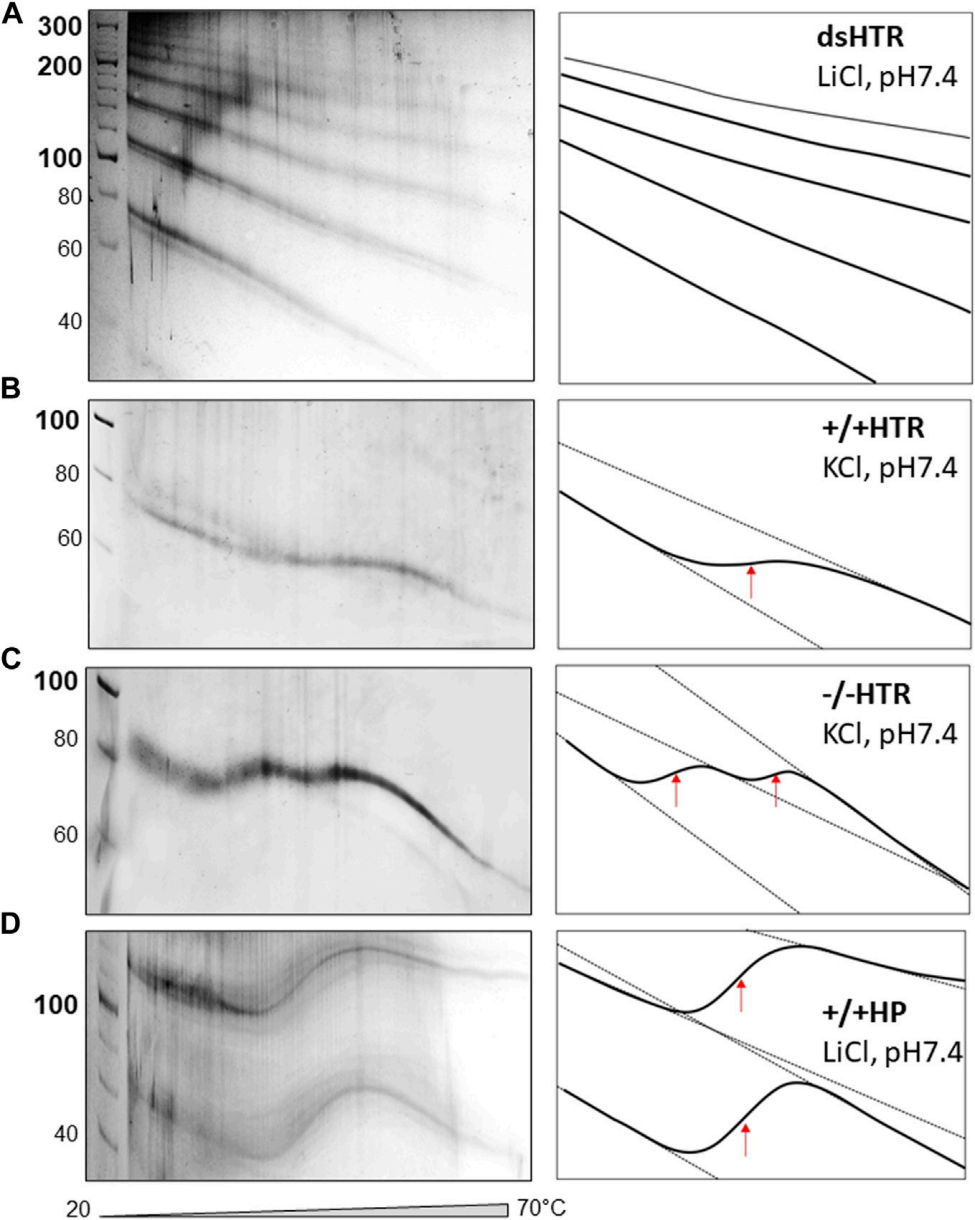
TGGE of MCs consisted of **(A)** dsHTR, **(B)** +/+HTR, **(C)** −/−HTR and **(D)** +/+HP. No significant change of linearity is detected for dsHTR, mobility linearly increases with increasing temperature. +/+ HTR is composed of two building blocks that can adopt G-quadruplex. Interestingly, −/− HTR able to adopt i-motif shows biphasic transition. The first transition occurs around 35°C and the second around 50°C.

The TGGE assays provided further indirect evidence to suggest that ligated building blocks form MCs and that the variable region can adopt NCMs (Figure 6). If no non-canonical motif is formed, then the MC mobility shows only a slight deviation from linearity, i.e., increasing mobility at increasing temperatures (Viglasky et al., 2000; Viglasky, 2013).

The electrophoretic observations of MCs consisted solely of perfectly complementary building blocks which displayed a characteristic signature (Figure 6A). However, if a non-canonical motif is formed and its stability is lower than that of the whole MC system, its melting can be captured by TGGE; deviation from linearity is a typical feature of this reaction. A specific S-shaped band was observed as the intramolecular G-quadruplex unfolds with increases in temperature (Viglasky et al., 2000). However, the unfolding of the G-quadruplex which forms part of MC was not detected by TGGE. It should be noted that the melting temperature was around 45 °C in the presence of 50 mM KCl, which is approximately 20 °C lower than the melting temperature of a DNA oligonucleotide with the same sequence under the same conditions (Bauer et al., 2010). This discrepancy can be explained by the fact that the HTR G-quadruplex embedded in MC adopts an antiparallel topology rather than a 3 + 1 hybrid structure. However, antiparallel HTR is usually formed in the presence of sodium, the stability of which corresponds to the results obtained under the given conditions.

The sequences capable of adopting i-motif structures offered some unexpected results, as a biphasic transition was observed; as can be seen in panel c, two clear distinct transitions were detected. Other authors have observed a similar effect, a biphasic transition earlier. However, this was only observed for C-rich sequences consisting of longer C-tracts capable of forming i-motifs (Školáková et al., 2019). However, this is not really our case. More experimental observations are needed to explain the mechanism of such a transition. A very clear transition was observed for a MC consisting of a sequence capable of forming hairpins which mimicked a cruciform structure (panel d).

## 4 Discussion

In general, the size of perfectly complementary MCs increases regularly with the number of building blocks that are ligated, but some oligonucleotides are capable of forming imperfectly complementary building blocks that form minicircles which are limited to a certain size. We assume that these defects are the cause of deformations in the basic building elements, such as a tendency to bend, and this is the reason why the formation of certain sizes of circles is unlikely. A similar effect has been observed for minicircles whose building blocks have been modified with platinum derivatives (Kašpárková et al., 1996).

The production of minicircles with customized DNA sequences, chemical functionalization and customized supercoiling could find further applications, including the study of interactions between minicircles and other molecules such as proteins and small ligands. For many reasons, the use of natural plasmids in this sphere is often very problematic. For example, our experimental observations have repeatedly confirmed that recombinant plasmids containing sequences capable of forming G-quadruplexes are consistently removed from the plasmids during cell cultivation by an unknown mechanism that has not yet been determined. Prokaryotic cells are known to use this unknown mechanism to remove some non-canonical motifs from plasmids, and it is assumed that other examples will be found in the future. However, this effect was not observed for recombinant plasmids featuring palindromic sequences capable of extruding the cruciform motif but only in cell types that had suppressed their repair.

The artificial plasmid mimicking system developed in our laboratory bypasses the problem of plasmid production by using cultivation. In principle, equivalent results were also obtained with building blocks containing d (CG)_n_ repeats at the cohesive ends instead of either d(A)_n_ or d(T)_n_, with these types of building blocks allowing MCs to be produced even at temperatures greater than 16°C (not shown).

## 5 Conclusion

The novel approach described in this study enables the quantitative production of a variety of DNA minicircles containing customized sequences capable of adopting different non-canonical motifs. The availability of such small DNA minicircles offers the opportunity to improve our understanding of the structural and dynamic diversity of DNA structural motifs and the ways in which they influence biological processes. The range of potential applications of MCs with non-canonical motifs are enormous; for example, they could function as carriers of a ligand that recognizes a specific non-canonical motif.

## Data Availability

The datasets presented in this study can be found in online repositories. The names of the repository/repositories and accession number(s) can be found in the article/Supplementary Material.
